# Moderate hypoxia induces metabolic divergence in circulating monocytes and tissue resident macrophages from Berkeley sickle cell anemia mice

**DOI:** 10.3389/fmed.2023.1149005

**Published:** 2023-07-12

**Authors:** Christina Lisk, Francesca Cendali, David I. Pak, Delaney Swindle, Kathryn Hassell, Rachelle Nuss, Gemlyn George, Pavel Davizon-Castillo, Paul W. Buehler, Angelo D’Alessandro, David C. Irwin

**Affiliations:** ^1^Cardiovascular and Pulmonary Research Laboratory, Department of Medicine, University of Colorado Denver–Anschutz Medical Campus, Aurora, CO, United States; ^2^Department of Biochemistry and Molecular Genetics, Graduate School, University of Colorado, Anschutz, Medical Campus, Aurora, CO, United States; ^3^Division of Hematology Colorado Sickle Cell Treatment and Research Center, School of Medicine, Anschutz Medical Campus, University of Colorado-Denver School of Medicine, Aurora, CO, United States; ^4^Department of Pediatrics, Hemophilia and Thrombosis Center, University of Colorado Anschutz, Medical Campus, Aurora, CO, United States; ^5^The Center for Blood Oxygen Transport, Department of Pediatrics, University of Maryland School of Medicine, Baltimore, MD, United States; ^6^Department of Pathology, University of Maryland School of Medicine, Baltimore, MD, United States

**Keywords:** sickle cell disease, hypoxia, spleen, pulmonary hypertension, metabolic disease

## Abstract

**Introduction:**

Human and murine sickle cell disease (SCD) associated pulmonary hypertension (PH) is defined by hemolysis, nitric oxide depletion, inflammation, and thrombosis. Further, hemoglobin (Hb), heme, and iron accumulation are consistently observed in pulmonary adventitial macrophages at autopsy and in hypoxia driven rodent models of SCD, which show distribution of ferric and ferrous Hb as well as HO-1 and ferritin heavy chain. The anatomic localization of these macrophages is consistent with areas of significant vascular remodeling. However, their contributions toward progressive disease may include unique, but also common mechanisms, that overlap with idiopathic and other forms of pulmonary hypertension. These processes likely extend to the vasculature of other organs that are consistently impaired in advanced SCD.

**Methods:**

To date, limited information is available on the metabolism of macrophages or monocytes isolated from lung, spleen, and peripheral blood in humans or murine models of SCD.

**Results:**

Here we hypothesize that metabolism of macrophages and monocytes isolated from this triad of tissue differs between Berkley SCD mice exposed for ten weeks to moderate hypobaric hypoxia (simulated 8,000 ft, 15.4% O2) or normoxia (Denver altitude, 5000 ft) with normoxia exposed wild type mice evaluated as controls.

**Discussion:**

This study represents an initial set of data that describes the metabolism in monocytes and macrophages isolated from moderately hypoxic SCD mice peripheral lung, spleen, and blood mononuclear cells.

## Introduction

Sickle cell disease (SCD) is a genetic hemoglobinopathy that disproportionally affects ethnic groups worldwide. In the US, approximately 1 out of every 365 African Americans and 1 out of 16,300 Hispanic Americans are born with this disorder (1). SCD is caused by a single point mutation in the HBB gene that results in expression and accumulation of hemoglobin S (HbS) in red blood cells (RBC) (2–4). Under hypoxic conditions, HbS is polymerized and forms insoluble fibers that are responsible for RBC sickling and vaso-occlusions. Although the genetic component of SCD is well defined, the disease progression manifests in a broad spectrum of hematological and vascular pathologies including pain, vision loss, leg ulcers, anemia, acute chest syndrome, splenic RBC sequestration, and pulmonary hypertension (PH) (5).

Advancements in metabolite analysis have created a new, unbiased, sensitive, high-throughput tool for scientists to investigate the etiology of SCD-related comorbidities at a molecular level and potentially inform on novel therapies. Tissue and cell specific metabolomics provide a versatile tool to help define and validate biomarkers for measurement of disease progression (6–10). Further, omics-based characterization of transgenic murine models of hemoglobinopathies provides a basic understanding of the altered metabolic state in murine SCD and beta thalassemia PH (11).

In the past decade it has become well recognized that dendritic cell and macrophage polarization contributes toward a wide range of pathophysiology in both the general and SCD patient populations, including PH (12–14). For instance, accumulation of pulmonary vascular macrophages is observed in the peripheral arterial adventitial sites of human SCD PH lungs at autopsy (12, 13). Our lab has shown that depletion of macrophages decreases both pulmonary vascular remodeling and right ventricular systolic pressures in a rat model of chronic Hb exposure and moderate hypoxia (8,000 feet, 15.4% O_2_) (12).

Macrophage metabolic reprogramming is associated with a pro- or anti-inflammatory state of tissue resident macrophages (e.g., lung, heart, spleen), with glycolysis, the Krebs cycle, and arginine/polyamine metabolism representing critical hubs regulating cell functional fates (15). Studies to date also suggest that cell origin dictates the metabolic reprogramming (at the transcriptional and functional levels) of resident versus recruited macrophages in the context of acute lung injury (16, 17). Thus, it is reasonable to suggest that macrophages in SCD have an altered tissue specific metabolic state and that studying these differences in tissues and blood will further define their role in PH development and other sequela of SCD.

In the current study macrophages were obtained from homozygous Berkeley (Berk) SCD mice following exposure to 10 weeks of moderate simulated hypobaric hypoxia to accelerate PH progression (18). Here, we test the hypothesis that the metabolism of lung and spleen tissue macrophages as well as PBMCs is altered in cohorts of Berk mice housed at either 8,000 feet (15.4% O_2_) or sea level (21% O_2_). The hypothesis is formed based on our studies that demonstrate chronic moderate hypoxia induces PH in Berk SCD mice that associates with sickling, accelerated erythrophagocytosis of injured RBCs and macrophage accumulation of iron (12, 19, 20).

## Materials and methods

### Animals

Aged matched (8 to 10 weeks old) female C57Bl/6 WT and Berk mice were either obtained from Jackson Laboratories (Bar Harbor, ME, United States) or our in-house Berk mouse colony. Mice were housed and bred in an AAALAC accredited animal facility at the University of Colorado, Denver, Anschutz Medical campus and were maintained on a 12:12 light–dark cycle with food and water available *ad libitum*. Female heterozygous Berk mice were bred with male homozygous Berk mice to generate homozygous offspring. Specifically, Berk mice with genotype Tg(Hu-miniLCR α1 ^G^γ ^A^γ δ β^s^) *Hba^0/0^ Hbb^0/0^* and the hemizygous with genotype Tg(Hu-miniLCR α1 ^G^γ ^A^γ δ β^s^) *Hba^0/0^ Hbb^0^ Hbb^+^* were littermates. Genotyping of mice used for breeding and experiments was performed by TransnetYX (Cordova, TN, United States). A total of 24 mice (n = 8 per group) were used to evaluate cardiovascular changes and a subset of 12 mice were utilized for metabolomics (WT: n = 6, Berk mice: n = 6) were used in the present investigation. Levels of discomfort and distress were monitored daily by the in-house animal care staff, with a veterinarian available as needed. Mice presented with no pain or discomfort associated with moderate hypoxia and were alert as well as eating, drinking, and grooming normally while housed. Hypoxia exposure consisted of mice housed at 8,000 feet (15.4% O_2_) for 10 weeks as previously described (11–13). All experimental procedures were conducted under the guidelines recommended by *The Journal of Physiology* (21), the National Institutes of Health and were approved by the Institutional Animal Care and Use Committee at the University of Colorado, Denver, Anschutz Medical Campus.

### Open chest solid state catheterization for right ventricular systolic pressure analysis

After 10 weeks in either normal or hypoxic environmental conditions, mice underwent terminal open chest right ventricular pressure measurements (RVSP) function measurements with a 1.2F, FTE-1212B-4,018 pressure volume catheter (Transonic Systems Inc., Ithaca, NY) inserted by direct cardiac puncture. Mice were induced inhaled isoflurane (4–5%), and tracheal incision (~1 cm) was performed. Next, a tracheal tube was inserted and connected to an Anesthesia Workstation or Hallowell EMC Microvent and an anesthetic plain was maintained at 1.0–2.5% isoflurane in 100% oxygen. After which, a thoracotomy was performed exposing the heart, the pericardium was resected, and a small hole made at the base of the right ventricle with a 30 g needle for insertion of the pressure-volume catheter. Steady state RVSPs, followed by contractility (Ees), and afterload (Ea) (during an occlusion by retracting the inferior vena cava), were captured and mice were humanely euthanized by exsanguination and cervical dislocation. Data was recorded continuously using LabScribe2 and analyzed offline.

### Peripheral blood mononuclear cell isolation

#### Peripheral blood mononuclear cell

Whole blood samples (1 mL) were obtained from animals *via* cardiac puncture, using a syringe with a 26-gage needle, and placed in an EDTA treated tube. The blood was transferred to a 15 mL conical tube and diluted 2:1, sterile PBS: blood, and gently mixed. Lympholyte® Mammal Cell Separation media (Cedarlane Labs, product # CL5115) was gently added to the bottom of the blood solution and spun at 1400 rpm for 30 min in a refrigerated centrifuge. After centrifugation, layers were visualized, the PBMC layer (midlayer) was extracted and resuspended in a new tube. The isolated PBMC’s were washed using ~14 mL sterile PBS, spun at 1800 rpm for 10 min, excess PBS was removed, cells were resuspended in 1-2 mL for counting, and the final pellet was frozen in liquid nitrogen and stored at -80C.

### Tissue macrophage isolation

#### Lung and spleen tissue macrophage collection

The right lung and spleen organ tissues were harvested, finely minced, and each organ placed individually in 2 mL Eppendorf tubes containing 1 mL of Collagenase D (Sigma Aldrich, product #11088866001) and DMEM media. The tissues were incubated at 37C with agitation for 30 min. After incubation, 100 μL of 0.1 M EDTA was added to the tissue-containing tubes and placed on ice. A single cell suspension was created by addition of Hanks buffered salt solution (HBSS, Corning, product #MT21022CV), passing through a 100 μm filter, and collected in a 15 mL conical tube. The tissue/cell suspension was spun at 500 g for 5 min and the supernatant discarded. The remaining tissue/cell solution was resuspended in 5 mL of RBC lysis buffer (Invitrogen eBiosciences, product #00–4,333-57), incubated at room temperature for 15 min, and centrifuged at 500 g for 5 min–this step was repeated if RBC presence was sustained. Next, the cells are washed with Miltenyi buffer (HBSS, 0.5 M EDTA, Fetal bovine serum), resuspended in 180uL Miltenyi buffer, and incubated on ice with CD11b Microbeads (Miltenyi Biotech, product #130–093-636) for 15 min. Positive cells were isolated using magnetic Ls columns (Miltenyi Biotech, product #130–042–401), collected in 2 mL Eppendorf tubes, counted, and then final pellet aliquots were frozen in liquid nitrogen and stored at -80C.

### Metabolomics

Lung and spleen macrophages and PBMCs were extracted in methanol:acetonitrile:water (5,3,2 v/v/v – at a 1 × 10^6^/ml and 10 mg/mL ratios) prior to UHPLC–MS analyses (Vanquish-QExactive, Thermo Fisher), as previously described.

### Statistical analysis

Hemodynamics: statistical comparisons for tissue and hemodynamic data measurements were completed with the analysis of variance (ANOVA) and *Post-hoc* analyses were completed with the Tukey–Kramer multiple comparison tests with statistical software package GraphPad (version 9.0). Other multivariate analyses, including hierarchical clustering analyses, heat maps, partial least square-discriminant analyses (PLS-DA), two-way ANOVA were performed with MetaboAnalyst 5.0 (22). Bar plots with superimposed dot plots were generated with Metabolite Autoplotter (23, 24). Data are presented as a mean ± standard error of the mean (SEM). Statistical comparisons for data measurements were completed with an *a priori* analysis using one tailed students *t* test for comparisons between wildtype vs. normoxic Berk and normoxic Berk vs. Berk hypoxia exposed. Statistical analysis was completed using the statistical software package GraphPad (version 9.1). Statistical significance was defined as *p* ≤ 0.05.

## Results

### Cardiovascular phenotypes are different in wild type and Berk mice exposed to moderate hypoxia

**Figure 1:**
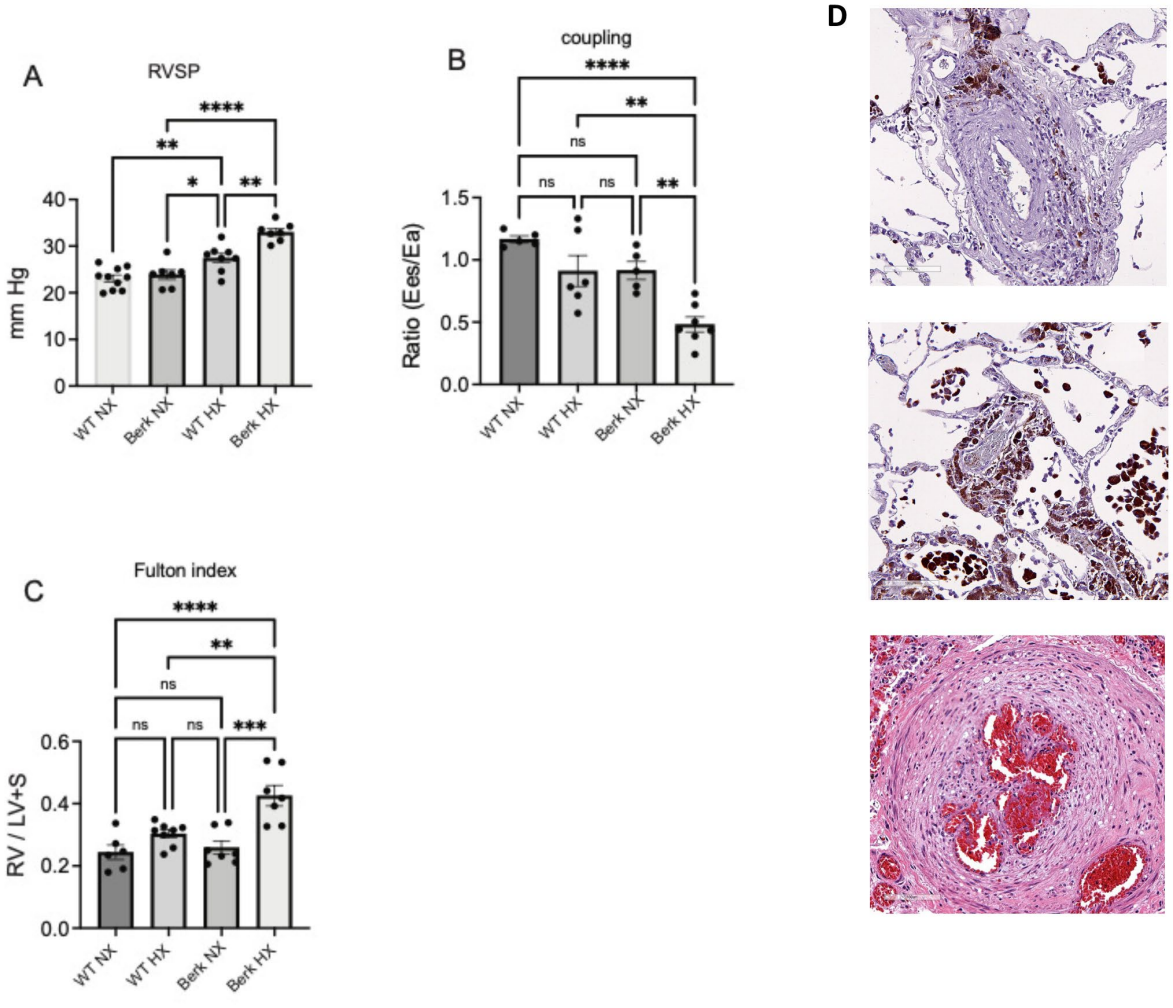
Cardiovascular phenotypes of wildtype and berk-ss exposed to hypoxia. **(A)** Right ventricular systolic pressures (RVSP); **(B)** Right ventricular to pulmonary vascular coupling ratio; **(C)** Right ventricular hypertrophy or Fulton index. *p<0.05, **p<0.005, ***p<0.002, ****p<0.001. RV-right ventricle; LV+S left ventricle plus septum; Ees- contractility; Ea- Afterload. **(D)** Pulmonary vascular changes in in human SCD-PH at autopsy: Top – peripheral lung vasculature macrophage iron accumulation in adventitia and Middle, iron loaded vascular and alveolar macrophages (tissue sections from patient reported in previous studies. Bottom image shows established plexiform lesion associated with advanced pulmonary vascular remodeling). Pathologies suggest consistency with results from Berk mice **(A-C)**.

To demonstrate differences in cardiovascular function between healthy wild type and Berk mice housed at sea level and moderate hypoxia, right ventricular systolic pressures (RVSP), ventricular to vascular coupling (ratio of contractility to afterload), and the Fulton index (RV/LV + S) were also compared between the four groups. At 20 weeks of age there was no differences in RVSP pressures (a reflection of pulmonary arterial pressure), ventricular vascular coupling ratio (contractility / afterload; a measure of energy transfer between heart and lung with a normal ratio ~ 1), and Fulton index (measurement of right ventricular hypertrophy) between the wild type (WT) and Berk Sea level cohorts (Figure 1). After 10 weeks of moderate hypoxia exposure Berk mice had higher RVSPs (27.44 ± 1 mm Hg wild type and 32.65 ± 1.1 Berk; *p* < 0.001; Berk hypoxia vs. wild type hypoxia; Figure 1A), lower ventricular to vascular coupling ratio (0.48 ± 0.06 Berk vs. 0.91 ± 0.07 wild type; *p* < 0.005; Figure 1B), and higher Fulton index (0.42 ± 0.03 Berk vs. 0.308 ± 0.013 wild type; *p* < 0.004; Figure 1C). As expected and congruent with our prior studies, these data demonstrate that unlike WT mice Berk mice exposed to moderate hypoxia develop a different pulmonary vascular phenotype more closely resembling active progression of pulmonary hypertension than wild type mice. These aberrant hemodynamic observations appear to be consistent with lung vascular pathology in human SCD-PH (Figure 1D).

### Metabolic phenotypes of PBMCs, spleen or lung macrophages are significantly different

**Figure 2:**
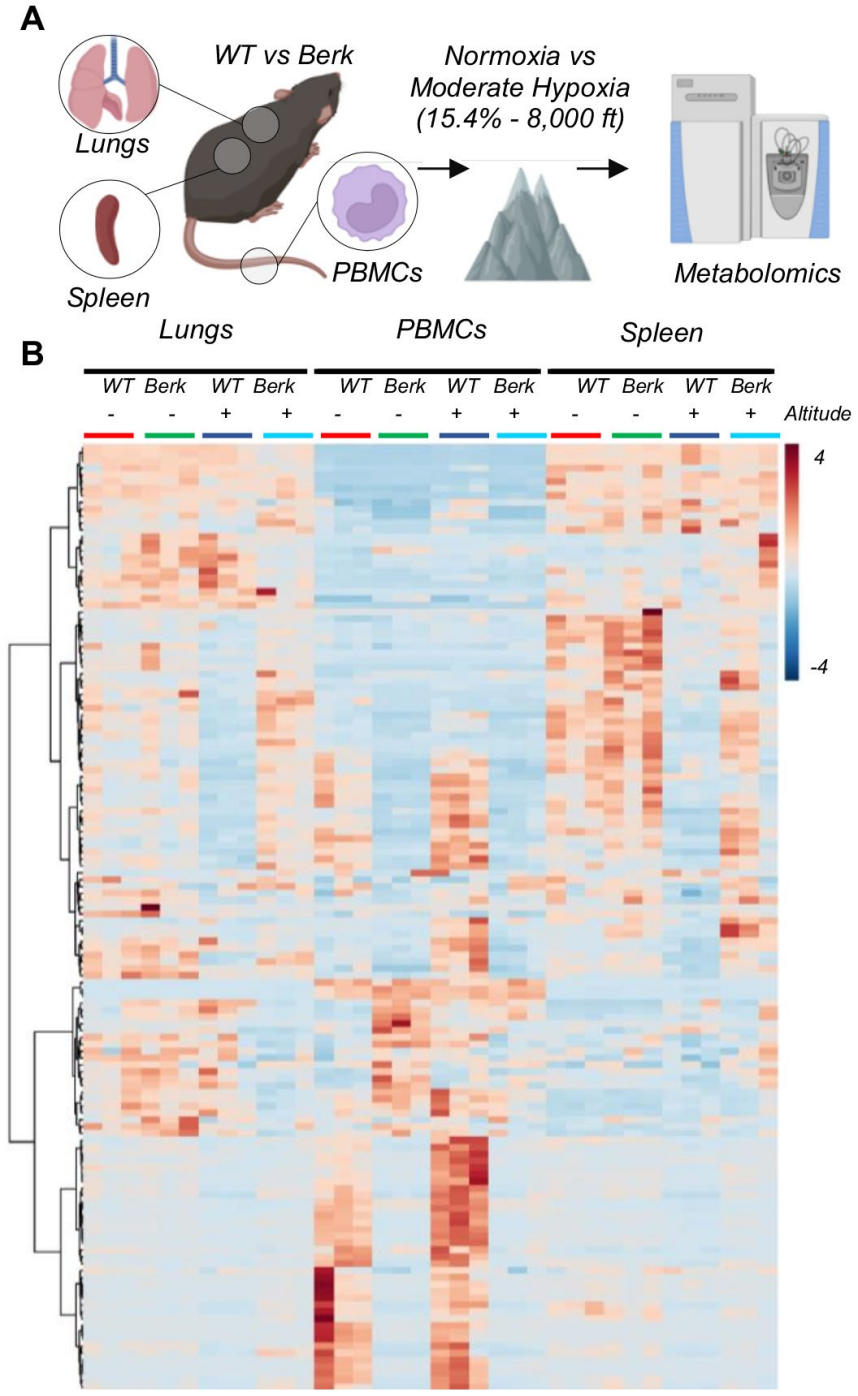
Experimental design for wild type and berk mice exposed to chronic hypoxia. **(A)** After 10-week exposure to either normoxic conditions or mild hypoxic conditions, peripheral blood mononuclear cells (PBMCs) and lung and splenic macrophages were isolated, and metabolomics were analyzed. **(B)** All metabolites analyzed from all samples.

Metabolomics analyses were performed on circulating PBMCs and resident splenic and lung macrophages (Figure 2A). Two-way ANOVA highlighted significant differences based on cell origin, with PBMCs clustering apart from resident macrophages. Hierarchical clustering analysis of the significant metabolites by ANOVA (Figure 2B) showed that the metabolic profiles of lung and spleen macrophages were overall comparable to each other, which was to be expected as they are fully differentiated resident macrophages. PBMCs from Berk mice were significantly different from WT PBMCs, while in resident macrophages differences emerged only upon 10-week exposure to moderate hypoxia (Figure 2B). Currently, it is unknown if splenic macrophages are recruited to the lung in PH pathogenesis and is a target of our ongoing studies.

### Glycolysis and Krebs cycle alterations in PBMCs from Berk mice in hypoxia

**Figure 3:**
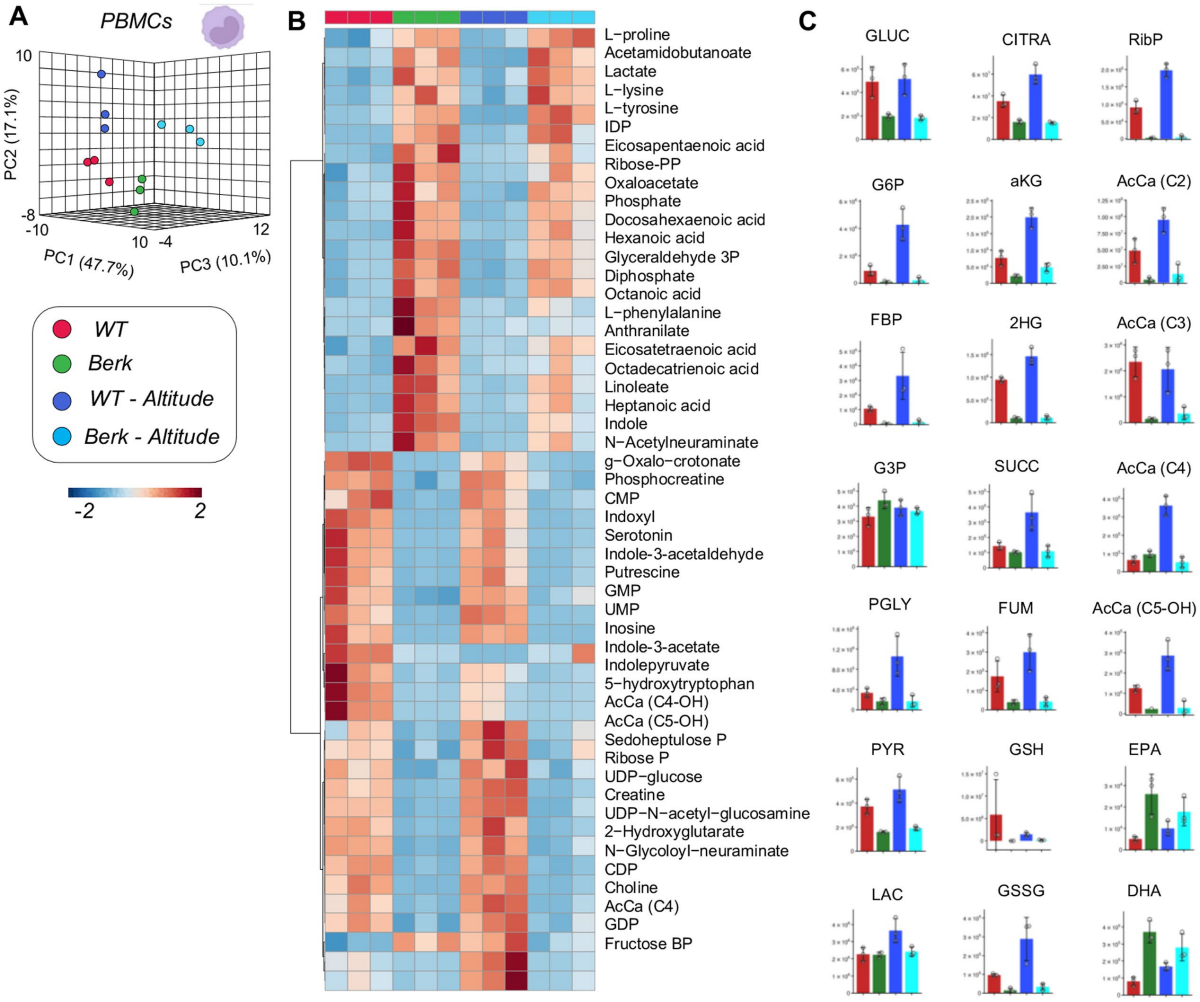
Metabolites from peripheral blood mononuclear cells (PBMCs). **(A)** 3D PCA analysis of PBMCs isolated from wildtype or Berk mice exposed to normoxic conditions or mild hypoxic conditions. **(B)** Top 50 metabolites via heatmap analysis. **(C)** Major metabolites from different metabolic pathways.

Focusing on PBMCs, we noted a significant difference between WT and Berk mice, which was evident by clustering across principal component 1 (explaining 47.7% of the total variance) in PLS-DA analyses (Figure 3A). An overview of the top 20 metabolites across PC1 is provided in Supplementary Figure 1 – mostly comprising of amino acids and free fatty acids. Altitude also had a lesser, albeit still significant effect on PBMC metabolism in both WT and Berk mice, effect that was captured by PC2 (17.1% of the total variance). In Figure 3B, we report the top 50 metabolites across the four groups as determined by two-way ANOVA. Metabolites in this list are enriched for glycolytic metabolites (fructose bisphosphate, lactate), amino acids (lysine, phenylalanine, proline, tyrosine – increasing in Berk mice), tryptophan metabolites (anthranilate, indole – increasing in Berk mice; serotonin, indoxyl, indole-acetate, indole-pyruvate – decreasing in Berk mice), free fatty acids (heptanoate, octanoate, linoleate, octadecatrienoate, eicosatetraenoate, eicosapentanoate, docosahexanoate – all increasing in Berk, but decreasing in hypoxia), acetyl-carnitines (AcCa C2, C4, C4-OH, C5-OH – increasing in hypoxia, only in WT). To further expand on these observations, we then focused on metabolites from the main energy-generating pathways, glycolysis and the Krebs cycle (Figure 3C). Results show that hypoxia promoted increases in the levels of all glycolytic metabolites in WT mice, but not in Berk mice. Elevated levels of carboxylic acids (including citrate, alpha-ketoglutarate – aKG, 2-hydroxyglutarate, succinate, fumarate) were observed in the hypoxic WT PMBCs, but not in Berk mice. Similar considerations can be made for short chain acyl-carnitines, all increasing in WT PBMCs upon exposure to hypoxia, but in Berk mice. Hypoxia was accompanied by consumption of reduced glutathione (GSH) and accumulation of the oxidized form (GSSG) in WT, but not in Berk mice.

### Spleen macrophages from Berk mice show distinct metabolic responses to moderate hypoxia compared to control mice

**Figure 4:**
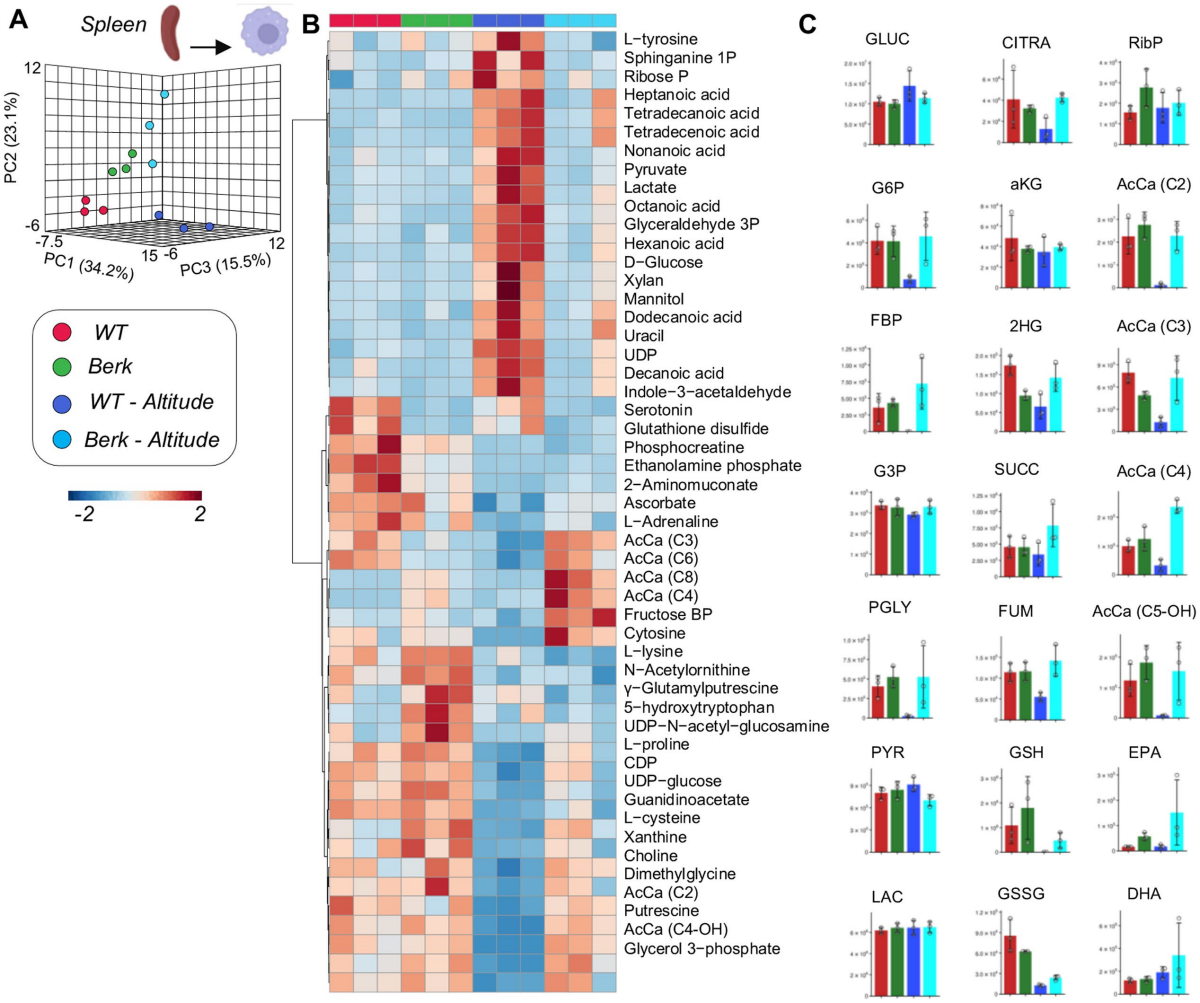
Metabolites from splenic macrophages. **(A)** 3D PCA analysis of splenic macrophages isolated from wildtype or Berk mice exposed to normoxic conditions or mild hypoxic conditions. **(B)** Top 50 metabolites via heatmap analysis. **(C)** Major metabolites from different metabolic pathways.

Metabolomics analyses of WT and Berk splenic macrophages following exposure to moderate hypoxia revealed a significant effect of reduced oxygen availability on metabolism (Figure 4A). Following exposure to hypoxia, splenic macrophages from WT mice, but not Berk mice, were characterized by lower levels of several glycolytic metabolites, including hexose phosphate, fructose bisphosphate, phosphoglycerate (Figures 4B,C). Hypoxia also promoted decreases in 2-hydroxyglutarate, fumarate in WT splenic macrophages, and decreases in reduced and oxidized glutathione in both WT and Berk macrophages. Berk mice were characterized by higher levels of multiple acyl-carnitines (C3, C4, C6, C8) in splenic macrophages (Figures 4B,C). Berk mouse splenic macrophages were characterized by higher (than WT) levels of highly unsaturated fatty acids (especially eicosapentaenoic and docosahexaenoic acid), a phenomenon that was exacerbated by hypoxia (Figure 4C). Together, these data highlight that wildtype mice have a rapid glycolytic flux, whereas the Berk mice rely on carnitine metabolism (an indication of pulmonary hypertension).

### Lung macrophages from Berk mice show distinct metabolic phenotypes in response to moderate hypoxia

**Figure 5:**
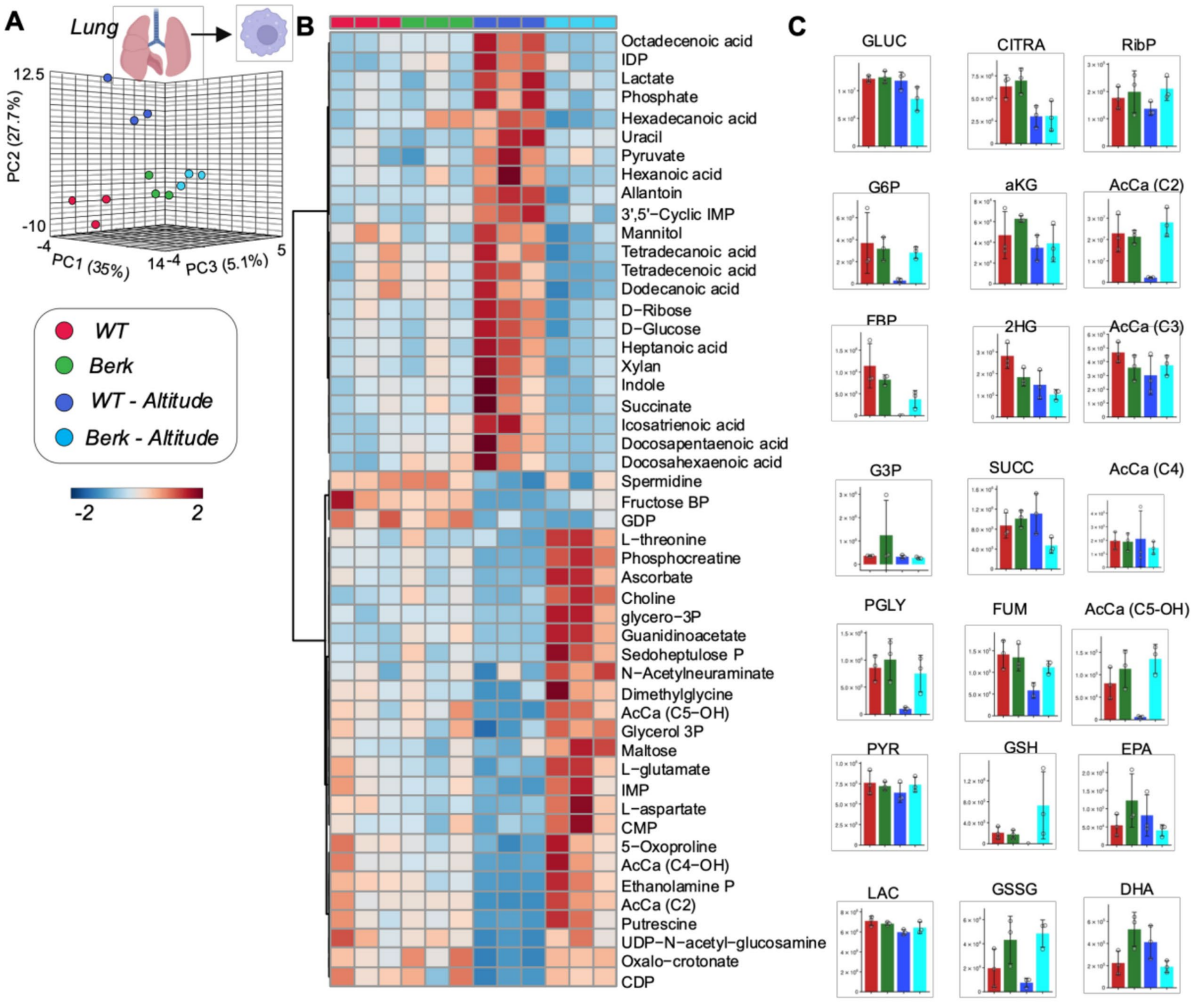
Metabolites from lung macrophages. **(A)** 3D PCA analysis of lung macrophages isolated from wildtype or Berk mice exposed to normoxic conditions or mild hypoxic conditions. **(B)** Top 50 metabolites via heatmap analysis. **(C)** Major metabolites from different metabolic pathways.

PLS-DA analysis of metabolomics data from lung macrophages showed distinct metabolic phenotypes between WT controls and Berk mouse lung macrophages, in normoxia and hypoxia (PC1 explaining 35% of total variance–Figure 5A). Hierarchical clustering analysis of the top 50 metabolites by two-way ANOVA highlighted significant increases in the levels of several saturated and monounsaturated fatty acids (C12, 14 and 16 series) in WT lung macrophages exposed to moderate hypoxia, compared to the other groups. Consistent with observations in splenic macrophages, lung macrophages were characterized by lower levels of multiple glycolytic metabolites following exposure to hypoxia (hexose phosphate, fructose bisphosphate, phosphoglycerate) and carboxylic acids (citrate, fumarate in Berk mice–Figure 5C). Unique signature in Berk mouse lung macrophages, reduced and oxidized glutathione levels were higher than WT counterparts at baseline and upon exposure to hypoxia (Figure 5B). Consistent with observations in splenic macrophages, hydorxyisovaleryl-carnitine (AcCa C5-OH) was the lowest in WT hypoxic lung macrophages (Figure 5C). To summarize, hypoxic exposure caused the WT lung macrophages mice to utilize the TCA cycle and fatty acids for energy. The berk mice, however, used amino acids and the glutathione pathway. The glutathione pathway is critical to maintaining redox homeostasis, which is disrupted in PH pathogenesis.

## Discussion

To date our studies in human and murine sickle cell disease (SCD) associate pulmonary hypertension (PH) have focused on hemoglobin (Hb), heme, and iron accumulation in pulmonary macrophages and their potential contributions toward progressive disease (11–13, 19, 25, 26). SCD is associated with increased incidence of PH and splenomegaly (27, 28). Although the development of PH is consistent with increasing age, the spleen is one of the most common and early organs to be affected in SCD (29-31). From a perspective of both PH and spleen function in SCD, it is recognized that macrophages are critically important in both the development of PH and processing of damaged red blood cells, respectively (12, 28, 34). While both the lung and spleen are capable of recruiting macrophages from the circulating monocyte pool, the metabolic differences between peripheral blood mononuclear cells (PBMC), lung, and spleen macrophages is unclear. In the current study, we hypothesized that the metabolomic profile of PBMCs, lung, and spleen macrophages would be altered in cohorts of Berk mice housed in normoxic (21% O_2_) and moderate hypoxia (8,000 feet equal to 15.4% O_2_). This hypothesis is formed on the basis that moderate hypoxia induces disease progression and accelerates erythrophagocytosis in Berk mice. To date we are unaware of any study that compares metabolomic profiles of circulating, lung, and spleen macrophages between healthy and SCD mice in normoxic and moderate hypoxic environments.

Confirming our previous work Berk mice housed in moderate hypoxia show increased RVSPs, ventricular to vascular coupling ratio, and RV hypertrophy that is consistent with the development PH associated with increased hemolysis (11, 19).

The metabolomic profiles of PBMC show dysregulation in amino acids, free fatty acids, and tryptophan metabolism in between Berk housed in both normoxic and hypoxic environments. Furthermore, moderate hypoxia alters the metabolomic profile in both strains of mice, demonstrating that even mild hypoxia can induce metabolic reprogramming of PBMCs. The metabolomic profile of PBMC populations in Berk mice show higher amino acids, free fatty acids, and tryptophan metabolites in comparison to wild type cohorts. Previous studies have shown that these pathways are all up-regulated in bone marrow-derived macrophages following erythrophagocytosis, *ex-vivo*. As such, the observation of elevated amino acid levels in Berk mice is consistent with phagocytosis of RBCs associated with hemoglobin polymerization, RBC sickling, and tissue damage from vaso-occlusion. Further, circulating PBMC are recruited to the lung and contribute to pulmonary vascular disease (11, 12). However, macrophages isolated from the lungs of wild type and Berk mice housed in either normoxia or hypoxia do not demonstrate a similar metabolic signature to PBMCs. This observation provides evidence to suggest that PBMC recruited to the lung may be metabolically reprogrammed within the pulmonary microenvironment. Although less clear, increases in free fatty acid and tryptophan metabolites, indole and anthranilate, suggest a macrophage driven process of inflammation (21, 35, 36, 37). Though speculative at this stage, it is interesting to note that these pathways are largely influenced by the interaction of blood and blood cells within the microbiome. Dysregulation of iron metabolism in SCD may thus affect siderophilic bacteria in the gut microbiome, and ultimately impact circulating cell levels of tryptophan metabolites and fatty acids.

Lung macrophages isolated from wild type and Berk mice housed in a normoxic environment show similar metabolic profiles. Compared to normoxic cohorts wild type mice exposed to moderate hypoxia showed both increases in glycolytic and fatty acid metabolites, consistent with hypoxia as a pro- inflammatory stressor. In contrast, lung macrophages in hypoxic Berk mice showed suppressed glycolytic metabolites, and increased pyruvate generation to meet the higher energy requirements caused from chronic exposure to hypoxia (21). Further, Berk mice exposed to hypoxia demonstrated higher ascorbate, and higher reduced and oxidized glutathione metabolites consistent with a hemolysis driven compensatory antioxidant processes (11, 19).

Splenic macrophages isolated from Berk mice exposed to hypoxia show a unique metabolomic signature consisting of higher levels of short (C3, C4) and medium (C6, C8) chain acylcarnitines. The well-established biologic function of acylcarnitines is to transport acyl groups from the cytosol into the mitochondria matrix for B-oxidation and sustain cell activity (38, 39). However, acycarnitines are increasingly identified as important indicators of metabolic disorders (38). It is possible that iron does alter mitochondria metabolism in iron overloaded macrophages. An equally plausible explanation is that altered iron metabolism or heme synthesis in SCD results in the dysregulation of catabolism of branched chain amino acids that fuel succinyl-CoA synthesis in mitochondria. This in turn results in the accumulation of short chain fatty acyl-carnitine metabolites (in equilibrium with their CoA counterparts). Notably, similar phenotypes are observed in other hemolytic disorders, such as pyruvate kinase deficiency or propionic acidemia.

In conclusion, this study was designed to address an initial understanding of how macrophages may contribute to PH, splenomegaly, vascular, or other metabolic disorders associated with SCD. To better understand mechanistic underpinnings of macrophage function in the sequela of SCD, future studies will focus on interrogating macrophages, analyzing metabolomic signatures and associating these findings with end organ injury and disease endpoints. The work presented herein provides a platform to expand on these concepts.

## Data availability statement

The raw data supporting the conclusions of this article will be made available by the authors, without undue reservation.

## Ethics statement

The animal study was reviewed and approved by Institutional Animal Care and Use Committee at Anschutz Medical.

## Author contributions

DI, AD’A, and PB: design of the work. CL, FC, DP, and DS: data collection. CL, DI, AD’A, and PB: Drafting the article. KH, RN, GG, and PD-C: critical revision of the article. All authors contributed to the article and approved the submitted version.

## Funding

The authors disclosed the receipt of the following financial support for the research, and publication of this article. This research was supported by NIH Grants RO1HL159862 (DI and PB), RO1HL152337 (DI and PB), RO1Hl161004 (DI, PB, and AD’A), RO1HL162120 (PB and DI), and K99HL156058 (PD-C).

## Conflict of interest

The authors declare that the research was conducted in the absence of any commercial or financial relationships that could be construed as a potential conflict of interest.

## Publisher’s note

All claims expressed in this article are solely those of the authors and do not necessarily represent those of their affiliated organizations, or those of the publisher, the editors and the reviewers. Any product that may be evaluated in this article, or claim that may be made by its manufacturer, is not guaranteed or endorsed by the publisher.

## Supplementary material

SUPPLEMENTARY FIGURE 1 Primary principal component top metabolites generated by MetaboAnalyst. Raw data was uploaded to MetaboAnalyst to analyze primary principal components in peripheral blood mononuclear cells (PBMCs, **A**), splenic macrophages **(B)**, and lung macrophages isolated from wildype and Berk mice in normoxic or hypoxia conditions. The top 30 significantly different metabolites are shown.
