# Supplementary figures and images for: Moderate hypoxia induces metabolic divergence in circulating monocytes and tissue resident macrophages from Berkeley sickle cell anemia mice
Source: Front Med (Lausanne). 2023 Jul 12;10:1149005. doi: 10.3389/fmed.2023.1149005 (PMC10370499; doi:10.3389/fmed.2023.1149005)

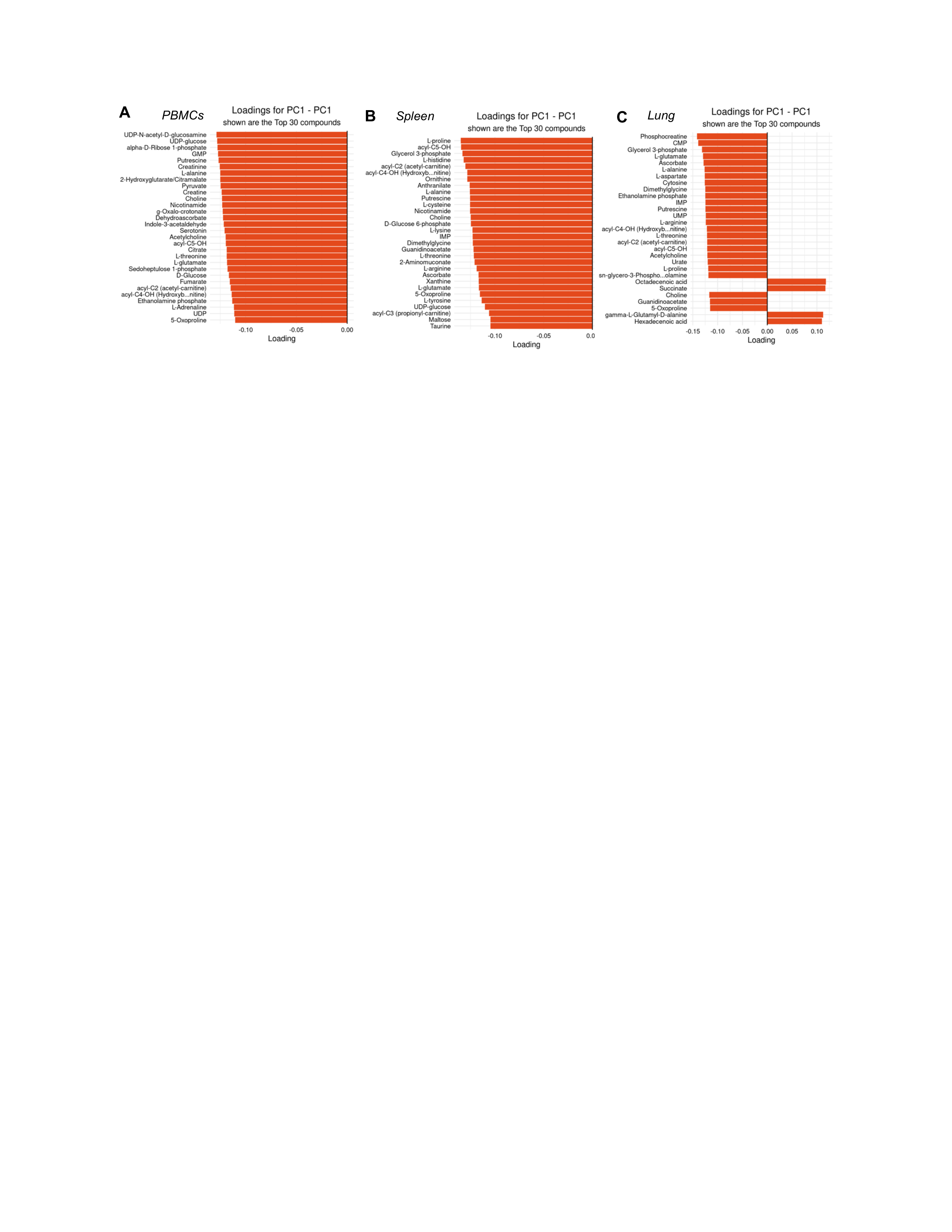

Supplement: SUPPLEMENTARY FIGURE 1 — Primary principal component top metabolites generated by MetaboAnalyst. Raw data was uploaded to MetaboAnalyst to analyze primary principal components in peripheral blood mononuclear cells (PBMCs, A), splenic macrophages (B), and lung macrophages isolated from wildype and Berk mice in normoxic or hypoxia conditions. The top 30 significantly different metabolites are shown. [file Image_1.tiff]
